# Development of an Innovative Optoelectronic Nose for Detecting Adulteration in Quince Seed Oil

**DOI:** 10.3390/foods12234350

**Published:** 2023-12-02

**Authors:** Saman Abdanan Mehdizadeh, Mohammad Noshad, Mahsa Chaharlangi, Yiannis Ampatzidis

**Affiliations:** 1 Department of Mechanics of Biosystems Engineering, Agricultural Sciences and Natural Resources University of Khuzestan, Mollasani 6341773637, Iran; 2 Department of Food Science & Technology, Agricultural Sciences and Natural Resources University of Khuzestan, Mollasani 6341773637, Iran; noshad@asnrukh.ac.ir; 3 Central Laboratory, Agricultural Sciences and Natural Resources University of Khuzestan, Mollasani 6341773637, Iran; m.4langi@gmail.com; 4 Southwest Florida Research and Education Center, Department of Agricultural and Biological Engineering, University of Florida, Gainesville, FL 32611, USA; i.ampatzidis@ufl.edu

**Keywords:** adulteration, colorimetric sensory, machine learning, volatile organic compound

## Abstract

In this study, an innovative odor imaging system capable of detecting adulteration in quince seed edible oils mixed with sunflower oil and sesame oil based on their volatile organic compound (VOC) profiles was developed. The system comprises a colorimetric sensor array (CSA), a data acquisition unit, and a machine learning algorithm for identifying adulterants. The CSA was created using a method that involves applying a mixture of six different pH indicators (methyl violet, chlorophenol red, Nile blue, methyl orange, alizarin, cresol red) onto a Thin Layer Chromatography (TLC) silica gel plate. Subsequently, difference maps were generated by subtracting the “initial” image from the “final” image, with the resulting color changes being converted into digital data, which were then further analyzed using Principal Component Analysis (PCA). Following this, a Support Vector Machine was employed to scrutinize quince seed oil that had been adulterated with varying proportions of sunflower oil and sesame oil. The classifier was progressively supplied with an increasing number of principal components (PCs), starting from one and incrementally increasing up to five. Each time, the classifier was optimized to determine the hyperparameters utilizing a random search algorithm. With one to five PCs, the classification error accounted for a range of 37.18% to 1.29%. According to the results, this novel system is simple, cost-effective, and has potential applications in food quality control and consumer protection.

## 1. Introduction

Quince seed oil (QSO) could be a wealthier source of dietary bio-components such as tocopherols, β-carotene vitamins, and cancer prevention agents; it is the foremost reasonable oil for human nutrition. Due to high prices and limited production, blending QSO with low-quality or low-price seed oils is becoming increasingly popular [1,2]. The adulteration of edible substances poses a significant contemporary food safety issue, with edible oils being among the most commonly adulterated food products. This form of adulteration presents a serious food safety concern, as it can result in both health risks for consumers and economic losses for producers [3]. As such, there is a pressing need for the development of various effective adulteration detection systems, each capable of identifying the presence of specific types of inferior ingredients in edible oils. Traditional methods of detecting adulteration, including chemical tests, chromatography, and spectroscopy, are often costly, time-consuming, and require skilled personnel [4]. Considering these limitations and growing concerns over food safety, the development of alternative technologies has become essential in the fight against food adulteration [5].

The practice of food adulteration consists of blending foods of lower nutritional value and prices. Often, the added oil is not easily detectable because additives do not alter general detecting indexes such as iodine value, saponification, and refraction index. In recent years, several nondestructive techniques have been developed and applied for edible oil adulteration detection, based on different physical principles and sensing modalities. These techniques offer advantages such as simplicity, speed, accuracy, low cost, portability, and online compatibility [6]. Mohan et al. [7] utilized the dielectric properties of extra virgin olive oil mixed with different adulterants (canola oil (46%), soy oil (17%), and castor oil (6%)) to detect differences between adulterated samples. The sensor demonstrated a fast response (100 ms) and good detection limits towards different adulterants, which made it suitable for high-throughput inspection. However, the results were susceptible to variations in electric current frequency and intrinsic factors (moisture, temperature, structural composition). Another research team employed low-field nuclear magnetic resonance relaxation fingerprints to rapidly identify adulteration of flaxseed samples by various concentrations of soybean oil [8]. The results indicated that adulteration in edible vegetable oils can be identified using the proposed method for the mixed flaxseed–soybean oils with adulteration ratios of 10% or greater. M. Shai et al. [9] developed a microwave planar resonant sensor for nondestructive detection of adulteration in olive oil mixed with mustard oil and sunflower oil. The results demonstrated a dependence of the sensor’s measured resonant frequency on the degree of adulteration in olive oil.

In recent years, electrochemical and colorimetric sensors, such as the Electrical Nose (E-Nose) and Chemical Nose (C-Nose), have gained popularity for detecting adulteration in food products [10]. A study conducted by Zarezadeh et al. [11] utilized an E-Nose in conjunction with machine learning techniques to classify and detect fraudulent activity in olive oil. Extra virgin olive oil was mixed with common frying oils, including sunflower oil, canola oil, and corn oil, to create six distinct classes for fraud creation at mass percentages of 5%, 10%, 20%, 35%, and 50%. The results of the study indicated a classification accuracy of 97.75%. Xu et al. [12] employed an E-nose in conjunction with cluster analysis, principal component analysis (PCA), and linear discriminant analysis methods to qualitatively analyze the oxidation of edible oils. The study successfully distinguished between oxidized and non-oxidized oils with accuracies of 95.8% and 98.9%, respectively. Huang et al. [13] utilized a colorimetric sensor array (CSA) to identify extra virgin olive oil adulterated with soybean oil and corn oil. The results of the proposed method were evaluated using linear discriminant analysis and successfully distinguished between the oils at different proportions. The accuracy of backtracking validation was 100% for both soybean oil and corn oil, while the accuracy of cross-validation was 90.7% and 81.5%, respectively.

This paper presents an innovative odor imaging system capable of detecting adulteration in quince seed edible oils based on their volatile organic compound (VOC) profiles. The system comprises a colorimetric sensor array, a data acquisition unit, and a machine learning algorithm for identifying adulterants. The system is simple, cost-effective, and has potential applications in food quality control and consumer protection.

## 2. Materials and Methods

### 2.1. Oil Sample Preparation

Quince seeds of the Isfahan variety were used to extract oil. After buying the quince seeds from the local market, the oil was extracted from the seeds using a mechanical pressing device, a BD oil cold press (Bekrdaneh Company, Isfahan, Iran). This was followed by several stages of seed oil filtration, which were carried out to remove solid impurities and contaminants. Pure and adulterated quince seed oil samples (containing different proportions of sunflower and sesame oil additives) were evaluated in this study. Six levels of adulteration (10%, 20%, 30%, 40%, 50%, and 100% or pure adulterant oil) and a total of 104 samples were assessed.

### 2.2. Sensor Array Development

**Figure 1:**
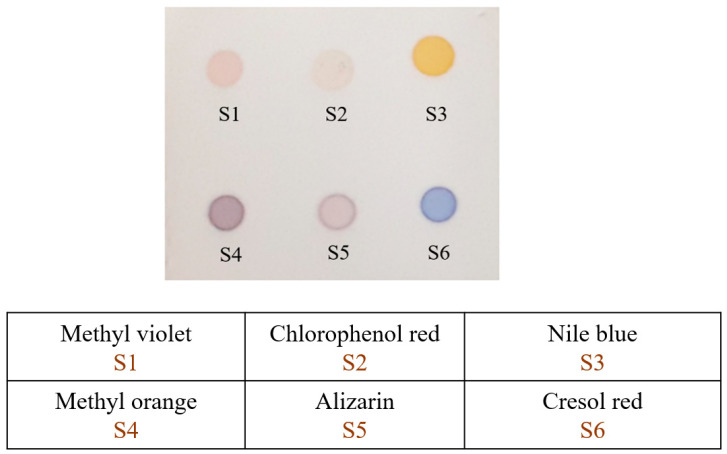
The fabricated colorimetric sensor array and the name of the chemical responsive dyes used in each cell array.

This sensor array was meticulously fabricated by depositing the solutions of six pH and redox indicators (as depicted in Figure 1) onto the surface of a Thin Layer Chromatography (TLC) plate (silica gel 60 F_254_ plate, Merck, Darmstadt, Germany). Each indicator was prepared by dissolving 5.0 mg of the indicator powder in 5.0 mL of either ethanol or deionized water. The resulting solutions were then mixed with tetrabutylammonium hydroxide (1.0 M in EtOH) in a ratio of 4:1 (Vindicator/VTBAH), which endowed them with hydrophobic properties, thereby preventing the dispersion of the indicator solutions on the substrate surface. A micropipette was employed to deposit a 1.0 µL aliquot of each indicator mixture onto the 2 cm × 2 cm TLC silica gel plate. The indicator spots (sensing elements) were placed approximately 5 mm apart on the TLC plate. Following fabrication, the sensor array was preserved in a desiccator for 12 h prior to subsequent utilization.

### 2.3. Color Information Extraction from Sensor Arrays

**Figure 2:**
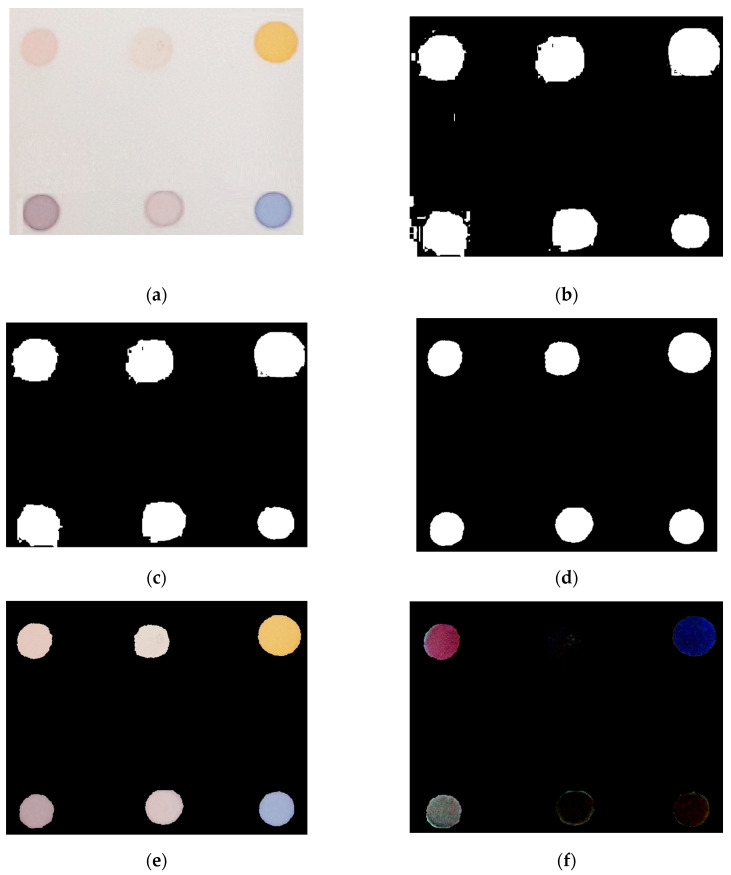
Image-processing steps for extracting color information from a sensor array: (**a**) original sensor array; (**b**) Otsu thresholding applied to the original image; (**c**) area-opening operation performed on the binary image; (**d**) erosion and area-closing operations applied; (**e**) binary image multiplied by the original image; (**f**) image after exposure to the volatile organic subtracted from images before exposure to the volatile organic.

In order to extract color information from the sensor array (Figure 2a), several image-processing techniques were employed. Firstly, the Otsu thresholding method was utilized to segment the sensor arrays from the background (Figure 2b) [14]. To eliminate any residual noise from the background, an area-opening operation was applied to regions smaller than 1000 pixels^2^ (Figure 2c) [15]. Due to suboptimal thresholding, the background surrounding the sensor array was not completely removed and some pixels within each sensor array were lost. To address this issue, erosion and area-closing operations were performed to remove the background surrounding the sensor array and to recover pixels within each sensor array, respectively. This ensured that redundant information was eliminated, and any lost pixel values were retained (Figure 2d). Each closed region was then labeled in a left-to-right manner, commencing from the upper part of the sensor base and progressing towards the bottom. The resulting binary image was multiplied by the color images, effectively isolating the sensor arrays from the background (Figure 2e). Next, the array sensor with the same label in the image prior to exposure to the volatile organic compound was subtracted from the corresponding array sensor following exposure to the volatile organic compound (Figure 2f). This image is a characteristic fingerprint of volatile compounds in oil samples. Finally, an analysis was undertaken with the objective of forming a feature vector. This was achieved by calculating the mean values of the RGB color channels (R, G, and B gray values) for each dye of the sensor array. With an array composed of six dyes, a total of 18 variables were consequently derived. Furthermore, to improve visualization, all color-difference maps contained herein are displayed by scaling an appropriate color range from a 6-bit (i.e., 3–62) to an 8-bit color scale (i.e., 0–255) [16].

### 2.4. Finding the Optimal Exposure Time for Sensor Array

**Figure 3:**
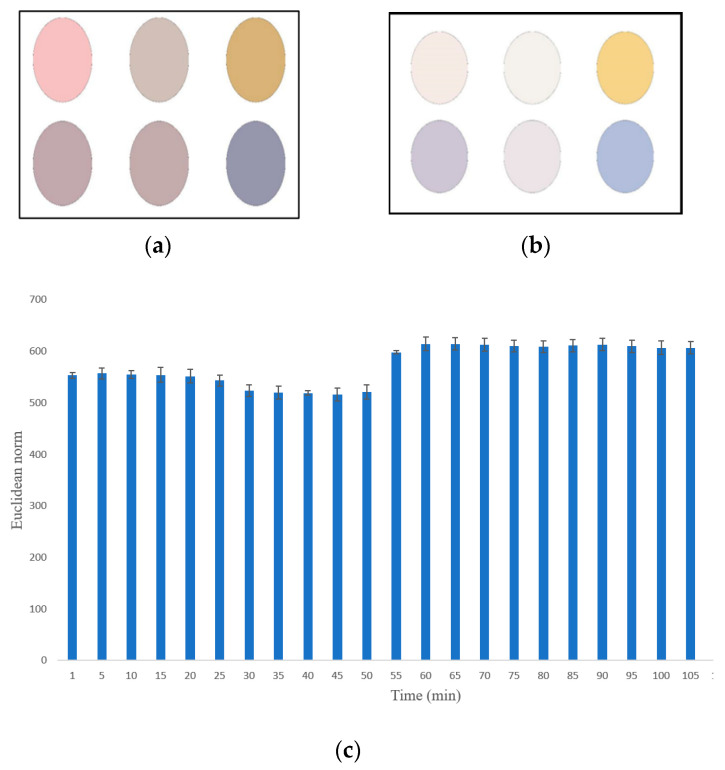
Sensor element response (**a**) prior to exposure; (**b**) subsequent to exposure to VOC; (**c**) color alterations observed over a duration of 120 min.

To determine the optimal exposure time for sensor response, a preliminary test was conducted. The sensor arrays were placed in the cap of a Petri dish and placed in an oven for a duration of 2 h at 60 °C. The volatile organic compounds (VOCs) extracted from oil samples were exposed to the surface of the sensor elements. During this period, a video was recorded and subsequently analyzed according to the methodology outlined in Section 2.3. The color differences were calculated at one-minute intervals and plotted (Figure 3). As depicted in Figure 3, the color changes reached a steady state after 60 min, with no further variation observed. Based on these results, this exposure time was selected for subsequent analysis. As explained previously, the response of the device to each sample is encapsulated by a data vector of a length of 18 units, each unit representing the mean values of the RGB color channels (R, G, and B gray values) for each of the six dyes in the sensor array. In the optimization step, the Euclidean norm of this vector was computed. The Euclidean norm, colloquially referred to as the ‘length’ of the vector, quantifies the magnitude of the vector. This was calculated as the square root of the sum of the squares of the vector components. This Euclidean norm of the 18-unit data vector was utilized as an analytical response. The condition yielding the vector with the maximal Euclidean norm was identified as the optimal condition.

### 2.5. Feature Selection and Classification

**Figure 4:**
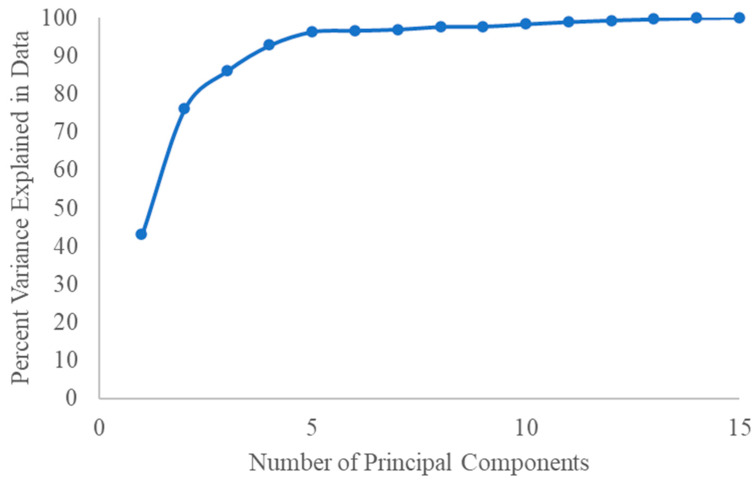
Percent variance explained by principal components.

Given that each dye is capable of providing three variables (R, G, and B gray values), a sensor array comprising six dyes can furnish a total of 18 variables. To extract the features, Principal Component Analysis (PCA) was applied to the data to reduce the dimensionality of the feature vector [17]. A plot illustrating the percent variance explained in data versus the number of Principal Components (PCs) was subsequently generated to ascertain the requisite number of PCs for classification (Figure 4). However, as depicted in Figure 4, five PCs accounted for over 95% of the variance in the dataset. Consequently, these five PCs were selected as inputs for classification, that is, the color information in this research. Nie et al. [18] implemented PCA on color information to reduce the high-dimensional data matrix and monitor the freshness of meat. Their findings indicated that a total of 10 PCs were necessary to account for 90% of the total variance, while 16 PCs were required to account for 95%. The authors attributed this to the high similarity in alkalinity and structure of the tested amine molecules.

The classification task was performed using the Support Vector Machine (SVM) algorithm. The SVM is a supervised machine learning algorithm that can be used for classification or regression tasks. It operates by finding the hyperplane that best separates the data into different classes. The hyperplane is chosen in such a way that it maximizes the margin between the classes. The margin is defined as the distance between the hyperplane and the closest data points from each class. These closest data points are called support vectors, hence the name Support Vector Machine. To optimize the classification performance of the SVM algorithm, a random search optimization algorithm was implemented [4]. This algorithm randomly samples values for the hyperparameters of the SVM algorithm, such as the kernel function, kernel scale, data standardization, and box constraint, and evaluates their performance. The best performing set of hyperparameters is subsequently selected [19].

The kernel functions evaluated comprised radial basis function (RBF), linear, and polynomial kernels. The kernel scale and box constraint were optimized within the range of [10^−3^, 10^3^]. This particular range is frequently employed in machine learning due to its ability to span several orders of magnitude. The breadth of this range facilitates the optimization algorithm’s search for the most effective parameters across both small and large scales, thereby enhancing the likelihood of identifying an optimal solution [19]. The box constraint parameter regulates the maximum penalty imposed on margin-violating observations. Margin-violating observations are data points that are on the wrong side of the margin or even on the wrong side of the hyperplane. By imposing a penalty on these observations, overfitting can be prevented. The kernel scale is a scaling parameter applied to the input data prior to processing by the kernel function. When the absolute values of certain features vary widely or are large, adjusting this parameter can prevent their inner product from dominating the kernel calculation. Data standardization involves centering and scaling each predictor variable by its corresponding mean and standard deviation. This can help improve the performance of the SVM algorithm by ensuring that all predictor variables have similar scales. The algorithm assessed both scenarios where data standardization was performed (true) and where it was omitted (false) to ascertain which approach yielded superior classification accuracy [20].

To obtain a better estimate of the classifier’s performance, a 10-fold cross-validation was conducted. The dataset was randomly partitioned into two subsets, with 70% (73 samples) utilized for calibration and 30% (31 samples) for prediction. This procedure was repeated ten times, each time selecting a different two-thirds of the dataset for calibration and the remaining third for prediction. The classification accuracy of the ten validation sets was then averaged and reported [21].

## 3. Results and Discussion

### 3.1. Sensor Array Interpretation

In the colorimetric sensor array methods, choosing the appropriate sensor elements is very important and effective on the results of measurements. The volatile organic compounds (VOCs) present in oil samples consist of acids, alcohols, aldehydes, and ketones. The VOCs differ among the oils, and hence they can be used to differentiate among the oils. According to the literature, these VOCs can interact with different pH and redox indicators that trigger a color change in the indicator. Therefore, we utilized a series of pH and redox indicators as sensing elements in the construction of this sensor array. Many reported studies confirmed that these VOCs can be interacted with indicators and lead to changes in their color intensity [22,23,24].

Since the base of chemical sensing is the intermolecular interactions of analytes and the sensor elements, then the type of these interactions is very important. These interactions consist of electrostatic ion–ion and proton acid–base interactions, hydrogen bonding, halogen bonding, charge-transfer and p–p molecular complexation, dipolar and multipolar interactions, and van der Waals interactions (e.g., physical adsorption). The main interaction in this sensor array is the acid–base interaction.

### 3.2. Adulteration Detection in Quince Seed Oils

The effectiveness of the fabricated colorimetric sensor array for identification of fraud in quince seed oil was thoroughly evaluated. This was achieved by adulterating quince seed oil with inexpensive oils such as sunflower oil and sesame oil at varying percentages. Since high levels of adulterants in quince seed oil samples can be visually discernible, the study focused on contamination levels below 50% for each adulterant. Pattern recognition methods, namely PCA and SVM, were employed to evaluate the discrimination ability of the developed sensor array.

**Figure 5:**
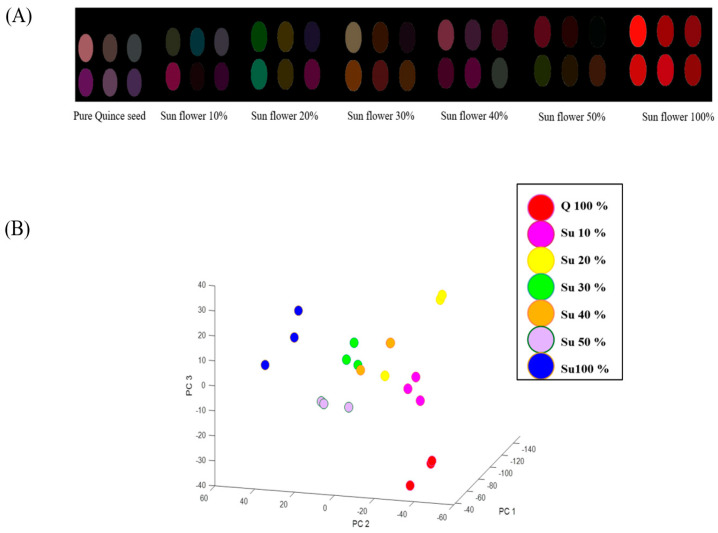
Color-difference maps (**A**) and the distribution of the samples in the 3-dimensional PCA score plot for quince seed oil contaminated with different amounts of sunflower oil (**B**). (“Q” represents quince seed oil and “Su” represents sunflower oil).

As per the findings, Figure 5A illustrates that the colorimetric patterns transition from pure quince seed oil to pure alternative oils as the volume ratio of sunflower oil (used as adulterants) is altered. The PCA score plot depicted in Figure 5B further corroborates the ability of CSA to discriminate between the binary mixtures of sunflower and quince seed oils.

In the detection of sesame oil adulteration in quince seed oil, different amounts of sesame oil (10%, 20%, 30%, 40%, and 50%) were introduced to quince seed oil. These adulterated samples were subjected to identical conditions as those of the quince seed oils adulterated with sunflower oils.

**Figure 6:**
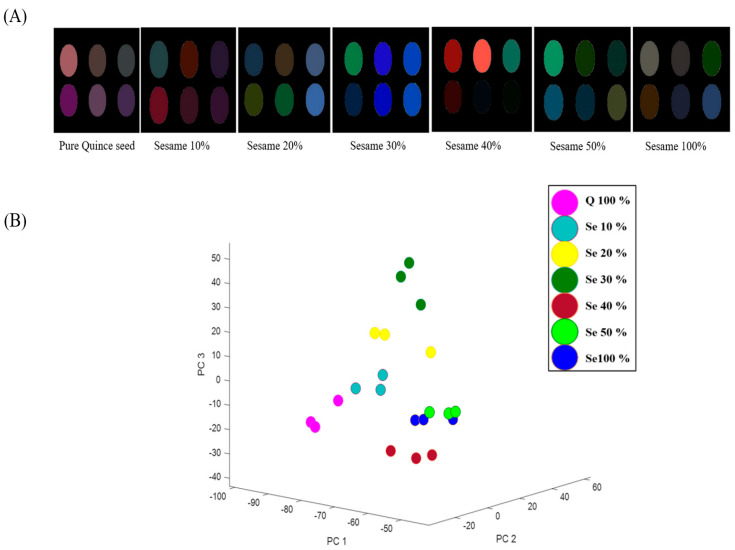
Color-difference maps (**A**) and the distribution of the samples in the 3-dimensional PCA score plot for quince seed oil contaminated with different amounts of sesame oil (**B**). (“Q” represents quince seed oil and “Se” represents sesame oil).

As illustrated in Figure 6, a noteworthy observation is the alteration in the colorimetric patterns from pure quince seed oil to sesame oil. It is evident that the color changes in methyl violet, methyl orange, and cresol red (sensors no. S1, S4, S6, respectively) in the color-difference map of pure quince seed oil differ from those containing substantial amounts (10%) of sesame oil. Moreover, oil samples adulterated with more than 20% sesame oil visually differ from pure quince seed oils for sensing elements of alizarine (S5). These results affirm the exceptional sensitivity of the proposed CSA in detecting trace amounts of sesame oil in the quince seed oil.

### 3.3. Classification Optimization and Accuracy

**Figure 7:**
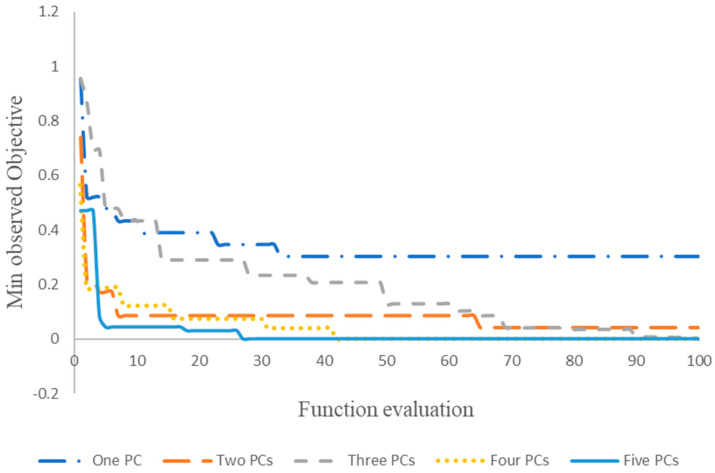
Minimum observed objective with one to five PCs.

A random search was performed to determine the optimal hyperparameters kernel function, kernel scale, data standardization, and box constraint. The minimum observed objective was plotted for 100 function evaluations with one to five PCs as inputs (Figure 7). The results showed that the objective function value decreased as the number of PCs increased. This value reached a plateau at 30.43% and 4.34% for one and two PCs, respectively, after 33 and 65 function evaluations. For three, four, and five PCs, the objective function value was zero after 99, 42, and 27 function evaluations, respectively.

Table 1 presents the optimized hyperparameters and the corresponding average of classification accuracy for the prediction set obtained with these optimal values. The results indicate that the classification error varies from 37.18 to 1.29 when using one to five PCs as inputs. However, no significant difference in the error rates is observed when three, four, or five PCs are employed. This could be attributed to the fact that the calibration step yielded zero error for the objective function, which is the classification error, when three to five PCs were utilized. In comparison, a study by Suslick [25] utilized a visual array sensor composed of four colorimetric sensors based on flavor-compound-targeted dyes and zinc ions as well as Data-Driven Soft Independent Modeling of Class Analogy (DD-SIMCA) as the classifier. The sensor array was exposed to different concentrations of sesame oil and adulterants such as soybean oil, corn oil, rapeseed oil, lard oil, cottonseed oil, and peanut oil. The method achieved a classification accuracy of 100% for both pure and adulterated sesame oil samples. Another study by Adade et al. [26] used surface-enhanced Raman spectroscopy (SERS) combined with chemometrics to detect the presence of Sudan dyes as adulterants in crude palm oil. Chemometric methods such as PCA, LDA, QDA, SVM and ANN were used to classify the samples. The best classification accuracy was obtained by SVM, with 98.75% for Sudan II and 97.5% for Sudan IV.

Table 2 presents the confusion matrix of the SVM classifier that was trained using five PCs. The results indicate that the classifier correctly classified all samples when sunflower oil was used as an adulterant. However, one quince seed oil sample adulterated with 10% sesame oil was misclassified by the classifier and assigned to the 20% sesame oil class. Despite this error, the sample was still identified as adulterated, indicating that the classifier was able to differentiate between sesame and sunflower oil adulterations even at low concentrations in quince seed oil samples. Kim et al. [27] analyzed the fatty acid composition of various vegetable oils, including sesame, quince seed, and sunflower oils. The analysis revealed that sesame oil and quince seed oil had similar fatty acid profiles, with both oils containing high amounts of oleic acid and linoleic acid. Conversely, sunflower oil had a different fatty acid profile, with a higher proportion of linoleic acid and lower amounts of oleic acid. Aghili et al. [28] used an E-nose to detect fraud in sesame oil mixture with sunflower oil using artificial neural networks (ANN). The electronic nose achieved a classification accuracy of 100% for pure sesame oil and 97.5% for adulterated samples. Moreover, the author stated that the proposed system could identify low-level fraud (25% sunflower oil to 75% sesame oil), which is difficult to detect using the GC-MS method, with very high accuracy by using the E-nose. Lu et al. [29] utilized an SVM classifier to identify extra virgin olive oil (EVOO) adulterated with other edible oils based on pigment compositions. The SVM classification of EVOO, other edible oils, and EVOO adulteration identification achieved 100% accuracy for the training set sample and 94.44% accuracy for the test set sample. However, the C-nose proposed in this study could identify lower levels of fraud (10% sunflower oil and 10% sesame oil) in quince seed oil. This suggests that the current approach has the potential to detect and quantify edible oil fraud more effectively and reliably.

## 4. Conclusions

In the study, a simple and low-cost optoelectronic nose was presented for the detection of fraud in quince seed oil samples, based on a sensitive colorimetric sensor array. This sensor was composed of a (2 × 3) array of pH and redox indicators. The color change profiles are individual fingerprints for each specific analysis and can be monitored with an ordinary flatbed scanner followed by unsupervised and supervised pattern recognition methods such as principal component analysis (PCA) and support vector machine (SVM). Furthermore, the study has shown that the color patterns produced were dependent on the type of adulterant, indicating the potential of this system for a wide range of applications. The use of machine learning algorithms enhanced the system’s ability to accurately identify adulterants, even in complex mixtures. The system’s simplicity and cost-effectiveness make it a promising tool for food quality control and consumer protection. It could potentially be used in various settings, from industrial food production to small-scale local producers, helping to ensure the integrity of food products and protect consumers from fraudulent practices. Moreover, the system’s adaptability suggests that it could be modified to detect other types of adulteration or contamination in different food products, further expanding its potential applications. Future research could explore these possibilities, as well as ways to further optimize the system’s performance. Overall, this study represents a significant step forward in the development of affordable, effective tools for food adulteration detection. It highlights the power of combining simple colorimetric sensors with advanced data analysis techniques to tackle complex real-world problems.

## Figures and Tables

**Table 1:** Results of optimization process as a function of PCs.

PCs	Box Constraint	Kernel Scale	Kernel Function	Data Standardization	Classification Error (%)
1	447.66	0.27376	Gaussian	True	37.18
2	0.001255	11.292	Gaussian	False	6.41
3	993.06	-	Polynomial of order 4	True	2.56
4	177.69	-	Linear	False	2.56
5	0.48491	-	Polynomial of order 2	True	1.28

**Table 2:** The confusion matrix of the SVM classifier.

	PQ	Su10:Q90	Su20:Q80	Su30:Q70	Su40:Q60	Su50:Q50	PSu	Se10:Q90	Se20:Q80	Se30:Q70	Se40:Q60	Se50:Q50	PSu
PQ	3	0	0	0	0	0	0	0	0	0	0	0	0
Su10:Q90	0	2	0	0	0	0	0	0	0	0	0	0	0
Su20:Q80	0	0	2	0	0	0	0	0	0	0	0	0	0
Su30:Q70	0	0	0	2	0	0	0	0	0	0	0	0	0
Su40:Q60	0	0	0	0	3	0	0	0	0	0	0	0	0
Su50:Q50	0	0	0	0	0	2	0	0	0	0	0	0	0
PSu	0	0	0	0	0	0	2	0	0	0	0	0	0
Se10:Q90	0	0	0	0	0	0	0	2	1	0	0	0	0
Se20:Q80	0	0	0	0	0	0	0	0	3	0	0	0	0
Se30:Q70	0	0	0	0	0	0	0	0	0	2	0	0	0
Se40:Q60	0	0	0	0	0	0	0	0	0	0	3	0	0
Se50:Q50	0	0	0	0	0	0	0	0	0	0	0	2	0
PSe	0	0	0	0	0	0	0	0	0	0	0	0	2

Note: “P” represents pure oil, “Q” represents quince seed oil, “Se” represents sesame seed oil, and “Su” represents sunflower seed oil. The number next to each abbreviation indicates the percentage of each oil present in the sample.

## Data Availability

The data presented in this study are available on request from the corresponding author.
